# Randomized Controlled Trial: Effects of a Bitter‐Tasting Pea Protein Hydrolysate Intervention With Low Degree of Hydrolyzation on Energy Intake in Moderately Overweight Male Subjects

**DOI:** 10.1002/mnfr.70195

**Published:** 2025-08-08

**Authors:** Katrin Gradl, Sonja Sterneder, Kristin Kahlenberg, Beate Brandl, Thomas Skurk, Veronika Somoza

**Affiliations:** ^1^ TUM School of Life Sciences Technical University of Munich Freising Germany; ^2^ Leibniz Institute for Food Systems Biology at the Technical University of Munich Freising Germany; ^3^ ZIEL ‐ Institute for Food & Health Freising Germany; ^4^ Chair of Nutritional Systems Biology Technical University of Munich Freising Germany; ^5^ Institute of Physiological Chemistry, Faculty of Chemistry University of Vienna Vienna Austria

**Keywords:** gastric emptying, human intervention study, pea protein hydrolysate, satiety, satiation

## Abstract

Optimizing plant‐based protein intake, such as pea protein hydrolysates (PPHs), may aid in obesity management. This study investigated whether PPHs with varying bitterness and degrees of hydrolysis (DH) differently affect satiety in healthy male participants. In a short‐term randomized control trial, 19 moderately overweight men (BMI 25–30 kg/m^2^) consumed boluses of 75 g glucose plus 15 g PPH (control without PPH; PPH1: less bitter, DH = 35%; PPH2: more bitter, DH = 23%). Upon PPH administration, energy intake from an ad libitum breakfast was reduced by −126 ± 329 kcal (*p* < 0.05) in the PPH2 group compared to the control. PPH1 decreased plasma ghrelin and DPP‐4 levels (AUC: −9.4 ± 19.6 and −12.5 ± 24.7, *p* < 0.05). Gastric emptying was delayed by a mean of 65% (*p* < 0.0001) after PPH2 consumption, assessed via ^13^C‐Na‐acetate breath test. Bitterness and DH of PPH influence satiety signals differently. PPH1 (less bitter, higher DH) reduces DPP‐4 and ghrelin levels, promoting satiety. PPH2 (more bitter, lower DH) delays gastric emptying, enhancing satiation. These findings highlight the potential of PPHs as functional ingredients in weight management strategies.

## Graphical abstract

Two pea protein hydrolysates (PPHs) reduced hunger by targeting different mechanisms: PPH1 produced a faster response in satiety and hunger hormone levels in the blood, while PPH2 had a stronger impact on gastric emptying. These effects are likely due to the differing degrees of hydrolysis between the two PPHs.

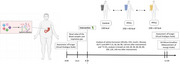

**Abbreviations**
5‐HT: serotonin
CCK: cholecystokinin
DH: degree of hydrolyzation
DOB: delta over baseline
DPP‐4: dipeptidyl peptidase 4
GLP‐1: glucagon‐like peptide‐1
HOMA: Homeostasis Model Assessment
PPH: pea protein hydrolysate
QUICKI: quantitative insulin sensitivity check index
VAS: visual analogue scale

## 1 Introduction

Obesity has emerged as a global health crisis, contributing to the rise of related diseases such as diabetes, cardiovascular disorders, and certain types of cancer [1]. Addressing this issue requires strategies that optimize diet to enhance satiety and reduce overall energy intake. A promising approach involves incorporating proteins, particularly plant‐based proteins, like pea protein, which not only serve as a sustainable alternative to animal proteins but also have the potential to induce satiation as well as longer‐lasting feelings of satiety [2, 3, 4, 5].

Satiation is the process that brings food intake to the end, driven by immediate physiological responses to the presence of food in the stomach and intestine [6]. Satiety, on the other hand, persists after a meal and determines how long it takes before hunger returns and the next meal is initiated [6]. Satiety is influenced by the macronutrient composition of the previous meal, with proteins being particularly effective at prolonging this sense of fullness [6]. Both processes are tightly regulated by hormonal signals, with proteins playing a significant role in enhancing these signals [5], helping control food intake and support weight management.

Neurotransmitters centrally regulate hunger and satiety [7] in the periphery by release of hormones from the gastrointestinal tract [7], where mechanisms such as gastric motility [8], gastric emptying, and gastric acid secretion [6] are controlled. Hunger increases as blood glucose levels drop and with the secretion of hormones such as ghrelin and motilin [7]. In contrast, satiety is mediated by an increase in blood glucose levels and the release of hormones like cholecystokinin (CCK), peptide YY, glucagon‐like peptide‐1 (GLP‐1), nitric oxide, insulin, and serotonin (5‐HT) [7]. In addition, other factors—including food composition, interactions with the microbiome, circadian rhythms, psychological influences, and sensory perceptions—play a significant role in regulating hunger and satiety [9].

Protein hydrolysates contain essential amino acids and are therefore important human nutrients, with their satiating effects well‐documented in both animal and human studies [2, 3, 4, 5]. Studies on satiety have shown that protein consumption and individual amino acids can increase feelings of fullness. In a study involving healthy men, Blom and colleagues [10] demonstrated that a high‐protein breakfast containing 57 g of wheat protein reduced the release of the hunger hormone ghrelin by a mean of 46% (*p* > 0.0001) compared to a high‐carbohydrate breakfast of equal caloric value. Furthermore, following a high‐protein breakfast (60% w/w protein), the plasma concentration of the satiety hormone GLP‐1 increased more (30%, *p* = 0.041) than after a high‐carbohydrate (60% w/w carbohydrate) breakfast [11]. Although animal proteins like casein or whey proteins have been predominantly used in the past, there is growing demand for plant proteins and their hydrolysates, for example, pea. This shift is mainly driven by the lower environmental impact of plant proteins, which require approximately 5–10 times less energy and water and reduce land use by about 80% [12]. Plant protein hydrolysates are an important source of essential amino acids and offer high nutritional value [13]. The pea protein hydrolysates (PPHs) currently available on the market are partial hydrolysates produced through various hydrolysis processes. Enzymatic or chemical hydrolysis of dietary proteins often generate a bitter, astringent off‐taste, which can limit their application in certain food products or require the addition of masking agents to improve palatability [14, 15]. From a nutritional point of view, increasing evidence suggests that bitter tasting food constituents targeting gastro‐intestinal bitter taste receptors (TAS2Rs) may regulate satiety mechanisms, such as delaying gastric emptying [16] and reducing energy intake by releasing satiety hormones [17, 18]. In a recent human intervention study by Stoeger et al. [18], it was shown that enriching a wheat protein hydrolysate with 3 g of the bitter‐tasting L‐arginine enhanced the satiating effect of the capsaicinoid nonivamide, leading to a decrease in energy intake (6.07 ± 4.38%, *p* < 0.01) and a slowing of gastric emptying (−2.10 ± 0.51%, *p* < 0.05), correlating with increased plasma concentrations of 5‐HT [15]. Similarly, a preceding study by Andreozzi and colleagues [17] showed that the bitter compound quinine reduces energy intake (−82 kcal, *p* = 0.007) in healthy individuals by increasing the postprandial release of CCK. Further, research by Wicks et al. [16] revealed that bitter taste affects gastric motility, as sham feeding bitter tastants to healthy female volunteers slowed down gastric motility.

In addition, research also highlights the pivotal role of the degree of hydrolysis in protein hydrolysates in modulating satiation and satiety, both through hormonal pathways and gastric emptying. Protein hydrolysates with a higher degree of hydrolysis tend to transit the stomach more rapidly, potentially resulting in faster gastric emptying [19] due to altered physicochemical properties like viscosity and foaming [20, 21]. In contrast, those with a lower hydrolysis degree require more time for gastric digestion. Moreover, more hydrolyzed proteins show stronger interactions with gut receptors, amplifying hormone release, shown in an increase in insulin release after ingestion of soy and whey protein compared to their hydrolysates [20, 22].

With this background, we aimed to investigate whether different PPHs vary in their impact on mechanisms controlling satiation and satiety to support the development of new generations of sensory‐attractive, satiating plant protein hydrolysates.

Optimizing dietary protein intake with plant‐based options like PPHs offers potential for reducing energy intake and mitigating obesity‐related risks. In addition, peas are one of the most promising plant‐protein sources with a rising market share [23]. Specifically, we studied two PPHs, differing in bitter taste and degree of hydrolysis, on their distinct short‐term satiating effects in a human intervention study with healthy male participants.

Specifically, we hypothesized that the more bitter‐tasting, less hydrolyzed PPH (PPH2) will have a stronger effect on satiation and satiety compared to the less bitter, more extensively hydrolyzed PPH1 by promoting the release of satiety‐related hormones influenced by its bitterness. Furthermore, the lower degree of hydrolysis in PPH2 may impact satiety by slowing gastric emptying, as its reduced hydrolysis is associated with slower digestion. Optimizing dietary protein intake, particularly with plant‐based options like PPHs, could be a powerful tool in helping to maintain a healthy body weight and combating obesity.

## 2 Materials and Methods

### 2.1 Study Design and Characteristics of the Participants

**FIGURE 1:**
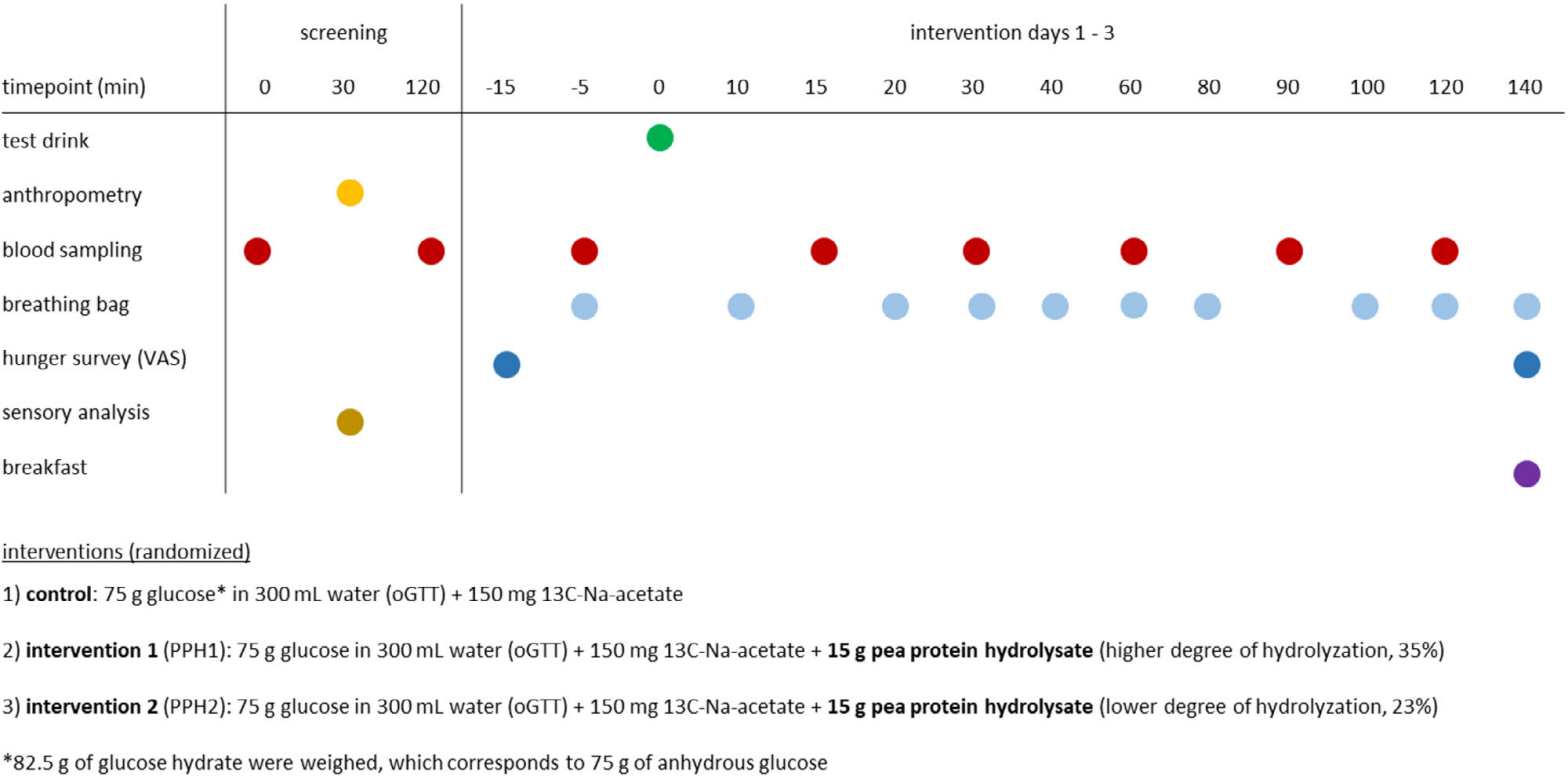
Study design of a randomized and single‐blinded human intervention study. A total of 19 volunteers underwent each of the three interventions with a 7‐day interval between interventions. For each day, blood samples and breathing gas were collected, and the subjective feeling of hunger by means of a visual analogue scale (VAS) was requested.

The study was conducted in agreement with the Declaration of Helsinki of 1975, as revised in 2013. The study procedure depicted in Figure 1 was approved by the Ethical Committee of the Faculty of Medicine of the Technical University of Munich (reference number 2023‐610‐S). An explanation of the risks, benefits, and the course of the study was given to each subject before the start of the study, and written informed consent was obtained from all participants. The protocol was registered in the German Clinical Trials Register (DRKS00033081). The characteristics of the test subjects can be seen in Table 1, and the CONSORT Flow Chart is depicted in Figure SI‐S5.

**TABLE 1:** Characteristics of the study participants.

Study subjects’ characteristics	
*N*	19
Sex	male
Age (years)	28.1 ± 5.6
Height (m)	1.80 ± 0.1
Body weight (kg)	87.8 ± 11.4
BMI (kg/m^2^)	27.1 ± 2.0
Fat mass (%)	24.0 ± 5.2
Fasting blood glucose (mg/dL)	92.9 ± 7.0

The study participants came to the study center on a total of three different days and received either the control, the more hydrolyzed PPH1 (PPH AMF Batch 221F007, degree of hydrolyzation (DH) = 35%, A. Costantino & C. S.P.A, Favria, Italy) or the less hydrolyzed PPH2 (Pea Peptone FM Batch 218/00221, DH = 23%, A. Costantino & C. S.P.A, Favria, Italy), blinded and in a cross‐over random order. The order of treatment administration was randomly assigned to the participants, ensuring that each possible sequence was represented equally across the study cohort. The manufacturer's stated degrees of hydrolysis were verified using LC‐ID‐TOF‐MS (Figure SI‐1). The differential bitterness of the two PPHs was previously assessed by a sensory trial, revealing that all panelists consistently described PPH2 as more bitter than PPH1 [24]. In addition, PPH1 and PPH2 were demonstrated to be degraded into bitter tasting peptides upon in vitro gastric digestion [24].

The G*Power 3.1.9.4 software was used to determine the number of study subjects. In a short‐term human study by Diepvens and colleagues [2], it was shown that the estimated interval between meals based on hunger scores in 39 healthy subjects was longest after a bolus of 15 g pea protein (117 and 151 min) compared to milk protein (100 and 141 min), whey protein (100 and 128 min) and the combination of whey and pea protein (95 and 131 min). A statistical error of *α* = 0.05 and *β* = 0.80 was used to calculate the number of subjects. This resulted in an actual power of 0.87 for a minimum subject number of 15.

A total of 19 male non‐smokers with regular eating habits completed the study. The subjects were selected according to the following inclusion criteria: male, aged between 18 and 45 years, BMI between 25 and 30 kg/m^2^, no alcohol or drug addiction, non‐smoker, no medication [18]. Disordered eating behavior was excluded from the Food Frequency Questionnaire of the Robert Koch Institute. To reduce biological variability, only male participants were included. Women were excluded due to cyclical fluctuations in estrogen levels during the menstrual cycle, which can affect peripheral 5‐HT concentrations and may confound outcome measures [25, 26].

Metabolic disorders were ruled out in advance using medical screening using a small blood count. Hematological parameters, plasma thyroid concentration, plasma triglycerides, and TNF were determined by a certified laboratory (Labor Becker MVZ eGbR, München). Furthermore, an oral glucose tolerance test (oGTT) was performed. After taking a baseline blood sample in the fasting state, volunteers received 75 g glucose. After 120 min blood was drawn and plasma glucose levels were determined (HemoCue Glucose 201+, plasma‐calibrated, Ängelholm, Sweden). All anthropometric and clinical parameters were measured in the morning following an overnight fast. Body composition and weight were measured using the Seca mBCA 515 scale (Seca GmbH & Co KG, Hamburg, Germany).

### 2.2 Test Day Protocol and Dosage Information

The test subjects received the following interventions in a random order on three study days after an overnight fast, applied as part of an oGTT [18]: Control (75 g glucose and 150 mg ^13^C‐Na‐acetate), higher hydrolyzed PPH1 (15 g PPH1, 75 g glucose and 150 mg ^13^C‐Na‐acetate), and a less hydrolyzed PPH2 (15 g PPH2, 75 g glucose and 150 mg ^13^C‐Na‐acetate). All three interventions were administered as beverages by dissolving the PPH powder, glucose, and ^13^C‐Na‐acetate in 300 mL of bottled, non‐carbonated Evian water, and were served in black opaque bottles. Before the intervention, volunteers were asked to rate their subjective feeling of hunger on a visual analogue scale (VAS) of 100 mm, followed by a breath sample and blood collection (Figure 1). Further blood samples were taken 15/30/60/90, and 120 min after administration of the oGTT [18]. In parallel, breath samples were collected every 10 min during the first 40 min, followed by the time points 60/80/100/120 and 140 min [18] (Figure 1). After the last breath sample, the subjective feeling of hunger was recorded again using VAS, and a standardized continental breakfast with ≈3000 kcal was served as ad libitum (Figure 1).

### 2.3 Blood Sample Collection

Venous blood was collected using an indwelling venous catheter 0, 15, 30, 60, 90, and 120 min after the intervention into EDTA tubes (Sarstedt, Numbrecht, Germany). The samples were immediately centrifuged at 2500 *g* for 10 min at 4°C and subsequently stored in aliquots at −80°C to determine 5‐HT, ghrelin, insulin, GLP‐1, CCK, and dipeptidyl peptidase 4 (DPP‐4).

### 2.4 Determination of Individual Caffeine Detection Thresholds

The individual bitterness threshold for each subject was determined according to ISO 3972 [27] by tasting caffeine solutions with concentrations between 0 and 0.27 g/L and evaluating the perceived bitterness. All test solutions were dissolved in Evian table water.

### 2.5 Determination of Plasma Glucose, Insulin, Ghrelin, CCK, GLP‐1, DPP‐4, and 5‐HT Concentrations

Glucose concentrations were measured colorimetrically using a HemoCue 201 system (Hitado, Möhnesee, Germany) at 0, 15, 30, 60, 90, and 120 min after the ingestion of the test drink. Insulin, ghrelin, 5‐HT, GLP‐1, DPP‐4, and CCK plasma concentrations were measured using an ELISA according to the manufacturer's protocol. The sandwich ELISA for insulin was purchased from Sigma Aldrich (Saint Louis, MO, USA). The competitive ELISA for GLP‐1 and CCK was obtained from Elk Biotechnology (Denver, CO, USA). 5‐HT samples were analyzed using the competitive 5‐HT ELISA (antibodies‐online, Limerick, PA, USA). To measure the ghrelin and DPP‐4 concentrations, the ELISA from R&D Systems, a Bio‐Techne brand (Minneapolis, MN, USA), and BT Lab (Shanghai, China) were used, respectively.

### 2.6 Gastric Emptying

The study beverage was labeled with ^13^C‐Na acetate (Euroisotop, Saarbrücken, Germany), which was prepared according to the guidelines for clinical trial materials. Breath bags for sample collection were obtained from FANci2 (Leipzig, Germany). Nineteen subjects per intervention provided breath samples in which ^12^CO_2_ and ^13^CO_2_ concentrations were quantified by infrared spectroscopy (FANci2, Leipzig). Specifically, each study participant had to fill a baseline breath bag (breath before the intervention) and then a breath bag at each time point (t10–t140). These bags were analyzed, and the device determined DOB (delta over baseline) values ([^13^CO_2_/^12^CO_2_]sample—[^13^CO_2_/^12^CO_2_]baseline sample) and the ^12^CO_2_ concentrations (%) of the sample and baseline as in previous studies [18].

### 2.7 Participants’ Rating of Hunger

Test subjects assessed their subjective feelings of hunger using a VAS both before and 140 min after the intervention. The VAS consisted of a 10 cm unstructured line, with 0 representing “not hungry at all” and 10 cm indicating “extremely hungry” [28, 29].

### 2.8 Energy Intake

The short‐term energy intake from the ad libitum breakfast [29] was analyzed by reweighing the remaining food on each subject's tray. The breakfast consisted of four rolls, three slices of whole grain rye bread, 3.5 slices of cheese (≈125 g), eight slices of ham (≈99 g), 80 g butter, 100 g jam, 60 g honey, 45 g coffee cream, 4 g sugar, 180 g yogurt, 200 mL water, and 200 mL coffee or tea [18]. Energy content and nutrient composition of the breakfast were calculated using OptiDiet Plus.

### 2.9 Statistics

All data are presented as mean ± standard error of the mean (SEM) unless otherwise indicated. As the two PPHs differed in their bitterness [24], which we hypothesized to influence satiety signals controlling food intake, the PPH treatments were matched for energy content to allow for a direct comparison. For each participant and each intervention day, all postprandial values were normalized relative to their respective fasting (baseline) value. This was done by calculating the ratio between the concentration at each postprandial time point and the fasting concentration: normalized value_(time)_ = concentration_(time)_/concentration_(fasting)_. To elucidate the specific effects of the PPH interventions, the normalized values obtained during the control intervention were subtracted from those of the PPH1 and PPH2 interventions at each corresponding time point: ΔPPH_(time)_ = Normalized value_(PPH,time)_—Normalized value_(control,time)_. Statistical analyses were performed using GraphPad Prism 10.2.1. Differences between the three treatments (control, PPH1, PPH2) were calculated by 2way ANOVA with Tukey's multiple comparisons test. Differences between the groups (control, PPH1, PPH2) for the mean delta at tx/t0 for all parameters were calculated using a 2way ANOVA with Tukey's multiple comparisons test for time points. For the comparison between the two PPHs (PPH—control) total area under curve (AUC) were calculated over time for plasma hormones and gastric emptying and tested between the two groups for statistical significance by conducting a Wilcoxon test. Different p values are indicated with asterisks according to the following scheme: *p ≤ 0.05, **p ≤ 0.01, ***p ≤ 0.001, and ****p ≤ 0.0001.

## 3 Results

### 3.1 Assessing the Subjective Feeling of Hunger by Means of VAS

Subjective hunger levels were measured using a 100 mm VAS both before and 140 min after the start of the oGTT. Comparing the interventions in a pre‐post format, the administration of PPHs reduced hunger compared to the control group. As shown in Figure SI‐2, participants reported an increased feeling of hunger following the control intervention (ΔVAS = +1.4 ± 0.6, *p* < 0.05). In contrast, hunger levels remained stable after the pea protein interventions (ΔVAS PPH1 = +0.5 ± 0.5, *p* > 0.05; PPH2 = +0.2 ± 0.5, *p* > 0.05), regardless of the specific PPH used.

### 3.2 Total Energy Intake After an Ad Libitum Breakfast

**FIGURE 2:**
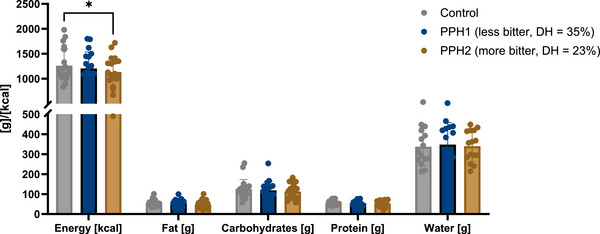
Mean changes in ad libitum nutrient and energy intake 2 h after the control, PPH1 and PPH2 interventions. Statistics: 2‐way ANOVA with Tukey's multiple comparisons test: ^*^ *p* ≤ 0.05. All data are provided as mean values with standard error of the mean (SEM) of *n* = 19 biological replicates.

A standardized breakfast was provided 2 h after administering the interventions to assess ad libitum energy intake. In the PPH2 group, a decrease of −126 ± 329 kcal in energy intake was observed (1262 ± 335 kcal for control vs. 1136 ± 316 kcal for PPH2, *p* ≤ 0.05, Figure 2), whereas no changes in ad libitum energy intake were found in the PPH1 group (1205 ± 330 kcal). Given that only the more bitter‐tasting and less hydrolyzed PPH2 led to a reduction in energy intake compared to the control, we aimed to explore differences in satiety signals between PPH2 and PPH1 to understand and explain this discrepancy in energy intake. Therefore, for further analyses, we normalized the PPH1 and PPH2 data to the control and compared the two groups directly.

### 3.3 Gastric Emptying

**FIGURE 3:**
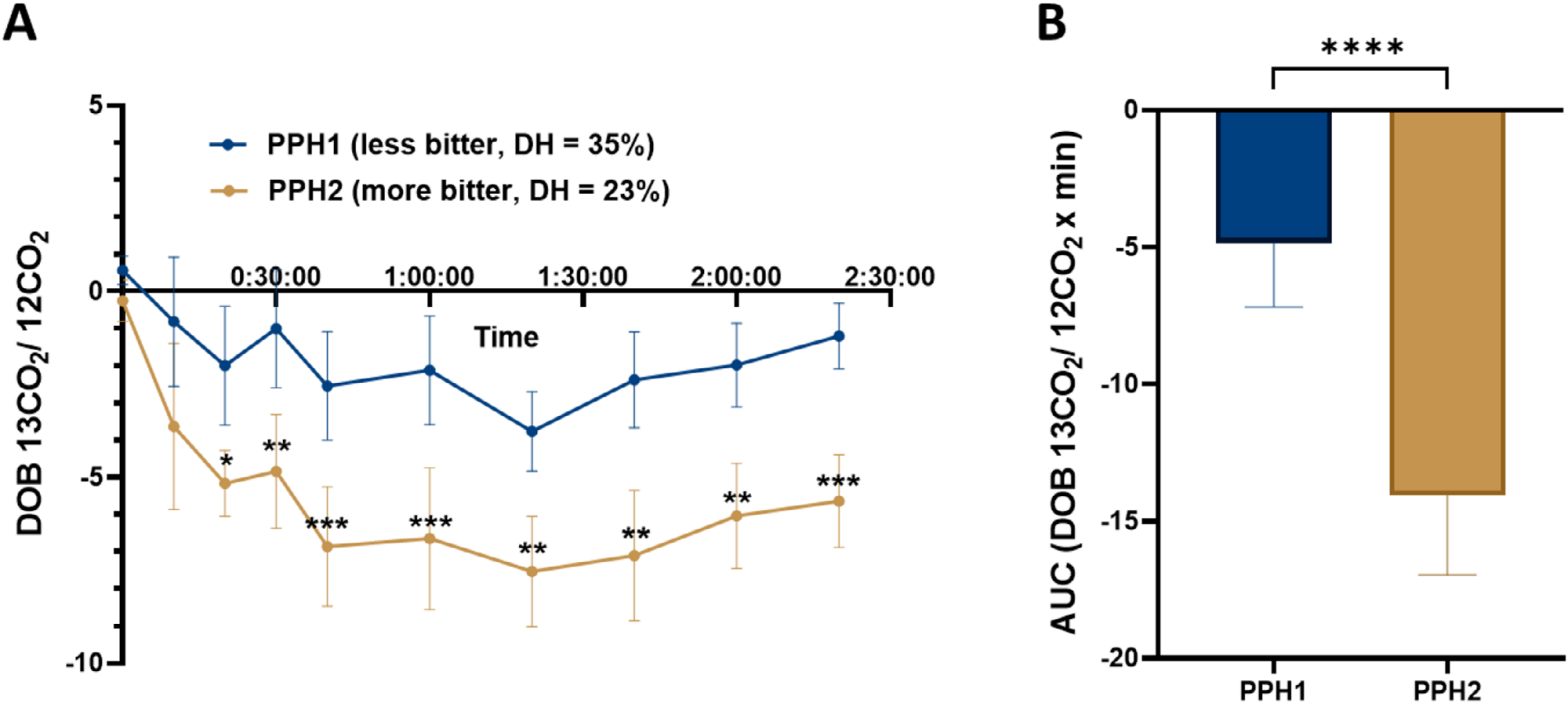
Comparison of gastric emptying after intervention with the hydrolysates PPH1 and PPH2 by calculating the ratio between labeled ^13^CO_2_ and naturally occurring ^12^CO_2_. Data shown as (A) mean value with SEM of 19 participants for each time point and (B) AUC of the time curves. Statistics: Wilcoxon test, significances are marked with ^*^ *p* ≤ 0.05; ^**^ *p* ≤ 0.01; ^***^ *p* ≤ 0.001; and ^****^ *p* ≤ 0.0001.

Analysis of the Area under the Curve (AUC) of the time curves for the 19 subjects revealed a significant delay in gastric emptying following the PPH2 intervention compared to the PPH1 treatment (AUC: PPH1: −4.8 ± 2.3, AUC PPH2: −14.1 ± 2.9; ΔAUC (PPH2‐PPH1): +9.21 = +65%, *p* < 0.0001; Figure 3B). In addition to the AUC analysis, a statistically significant delay in gastric emptying was observed with PPH2 compared to PPH1 at time points t20 to t140 (Figure 3A).

### 3.4 Plasma Concentrations of Glucose, Insulin, 5‐HT, CCK, and GLP‐1

The comparison between the two hydrolysate interventions revealed no significant differences in the mean plasma levels of glucose and insulin, and the calculated Homeostasis Model Assessment (HOMA) index and quantitative insulin sensitivity check index (QUICKI) (Figure SI‐3), as well as 5‐HT, CCK, and GLP‐1 (Figure SI‐3) following oral glucose administration. The mean plasma concentrations remained similar over time, and analysis of the AUC between the treatments confirmed this lack of statistical significance.

### 3.5 Plasma Ghrelin Concentrations

**FIGURE 4:**
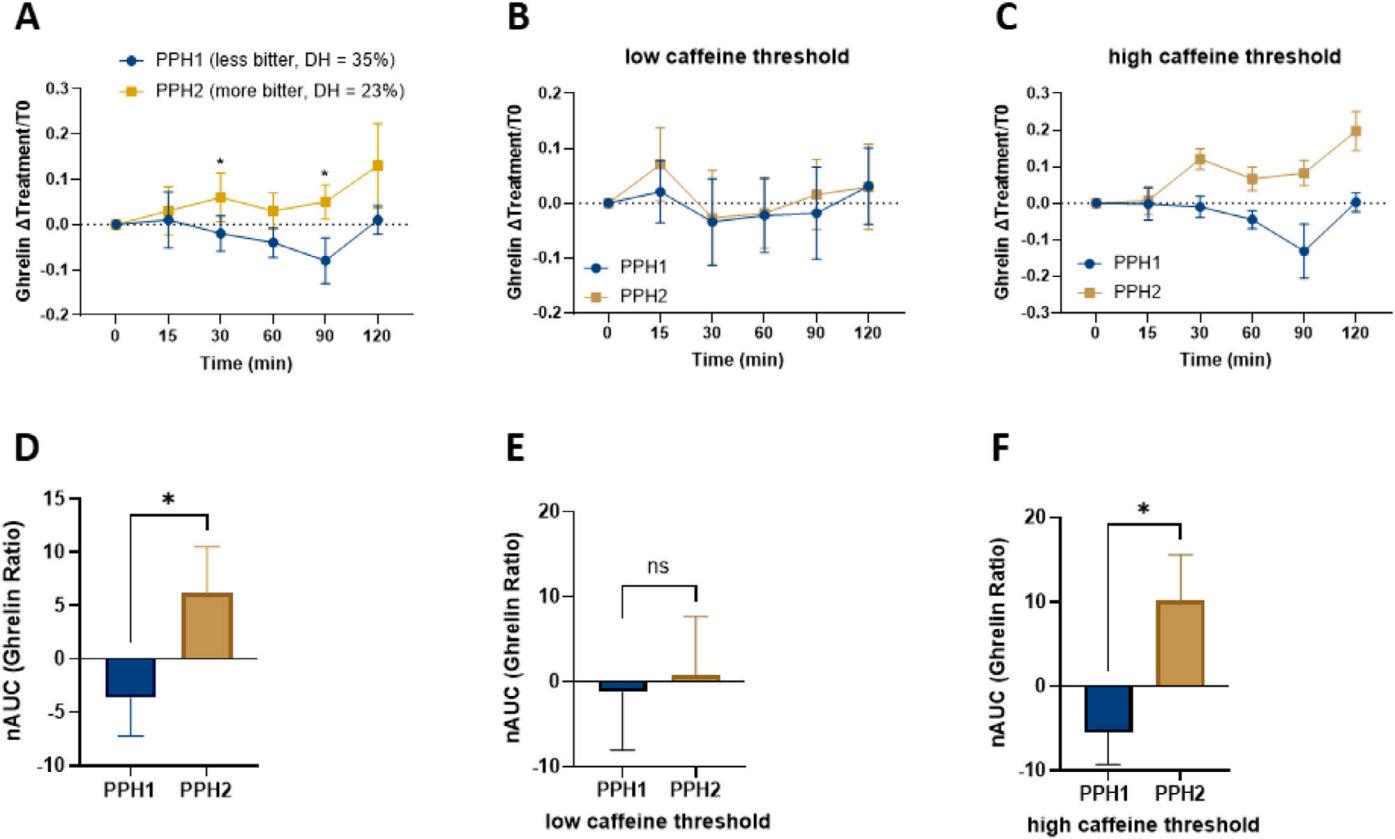
Comparison of ghrelin blood plasma levels after intervention with the hydrolysates PPH1 and PPH2 by calculating the ratio between the fasting ghrelin concentration and each time point. Data are shown as (A) mean value with SEM of 19 participants for each time point and (C) AUC of the time curves. Additional comparison of ghrelin levels between low and high caffeine detection threshold groups, calculating the ratio of fasting ghrelin to each time point. Data are presented as follows: Mean values with SEM of 19 participants at each time point in the low (E) and high caffeine threshold groups (F) and AUC for ghrelin in the low (B) and high caffeine threshold groups (C). Wilcoxon test, significance values are marked with *p ≤ 0.05.

To assess short‐term satiety, plasma ghrelin concentrations were measured both in the fasting state and at multiple time points after the oGTT (t15, t30, t60, t90, and t120). At baseline, no significant differences were observed in mean plasma ghrelin levels between the PPH1 and PPH2 interventions. But notably, lower ghrelin levels were detected in the PPH1 group at both 30 and 90 min (*p* < 0.05, Figure 4A) compared to PPH2. In addition, the AUC for ghrelin was 150% lower (*p* < 0.05) following the PPH1 treatment (−3.7 ± 3.6) compared to the PPH2 intervention (6.2 ± 4.3) (Figure 4D). The difference in ghrelin levels between PPH1 and PPH2 in blood plasma is primarily driven by the high caffeine detection threshold group (detection threshold ≥0.14 g/L), as a significant difference in AUC was observed exclusively in this group (Figure 4B–F).

### 3.6 Plasma DPP‐4 Levels

**FIGURE 5:**
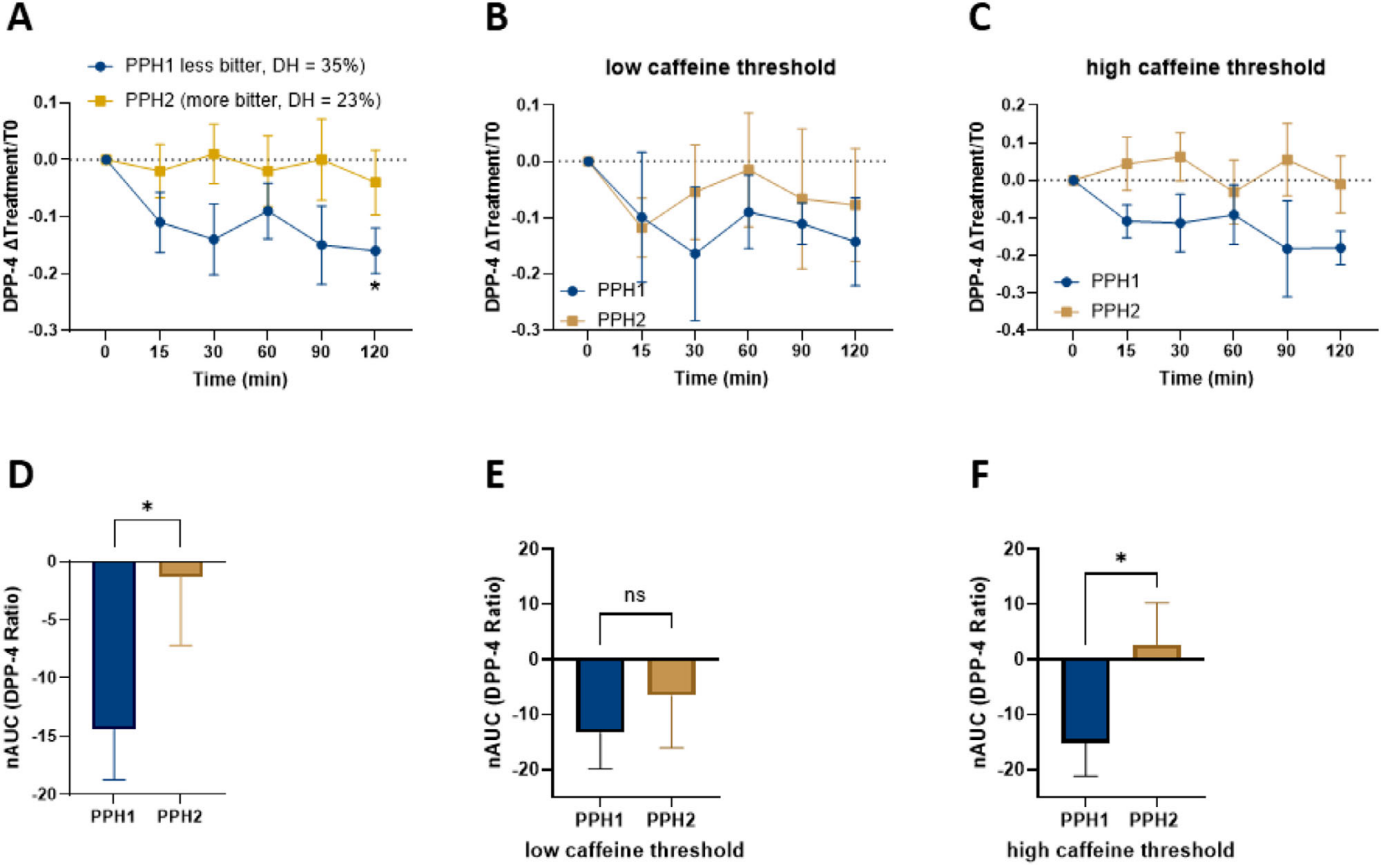
Comparison of DPP‐4 blood plasma levels after intervention with the hydrolysates PPH1 and PPH2 by calculating the ratio between the fasting DPP‐4 concentration and each time point. Data are shown as (A) mean value with SEM of 19 participants for each time point and (C) AUC of the time curves. Additional comparison of DPP‐4 levels between low and high caffeine detection threshold groups, calculating the ratio of fasting DPP‐4 to each time point. Data are presented as follows: Mean values with SEM of 19 participants at each time point in the low (E) and high caffeine threshold groups (F) and AUC for DPP‐4 in the low (B) and high caffeine threshold groups (C). Wilcoxon test, significance values are marked with *p ≤ 0.05.

To assess another satiety parameter, plasma DPP‐4 concentrations were measured both in the fasting state and at various time points after the oGTT (t15, t30, t60, t90, and t120). Similar to ghrelin, no significant differences in mean DPP‐4 levels were observed between the PPH1 and PPH2 interventions at baseline. However, after 120 min, lower DPP‐4 concentrations were recorded in the PPH1 group compared to the PPH2 group (ΔPPH1‐PPH2 = −0.12 ± 0.19, *p* < 0.05, Figure 5A). Additionally, the AUC for DPP‐4 was reduced following the PPH1 treatment (−14.4 ± 4.3, *p* < 0.05) compared to the PPH2 intervention (−1.3 ± 5.9) (Figure 5D). For DPP‐4 levels, a similar pattern is observed as with ghrelin levels, with a difference in AUC between the PPH1 and PPH2 interventions detected only in the high caffeine detection threshold group (Figure 5B–F).

## 4 Discussion

Consuming foods rich in components that enhance satiation and satiety offers a promising strategy to reduce daily caloric intake and combat the growing issue of obesity. This study aimed to evaluate whether a more bitter, less extensively hydrolyzed PPH (PPH2) exerts a stronger effect on satiety and satiation than a less bitter, more hydrolyzed variant (PPH1). Additionally, it sought to determine if any differences in satiety could be attributed to distinct mechanisms of action.

We hypothesize that the more bitter, less hydrolyzed PPH2 will stimulate the release of satiety hormones due to its more intense bitterness. Additionally, we expect it to have a greater effect on delaying gastric emptying, as it requires more extensive gastric digestion to break down the hydrolysate.

In this study, total energy intake and satiety‐related gastrointestinal and peripheral signals were evaluated following the administration of a caloric load (75 g glucose as a control) in combination with either 15 g of PPH1 (less bitter, higher degree of hydrolysis) or 15 g of PPH2 (more bitter, lower degree of hydrolysis). The bolus dose of the PPH was chosen based on a short‐term human study by Diepvens and colleagues [2], which compared the satiating effects of various protein sources. The two PPHs were selected for their pronounced differences in their bitter taste and hydrolysis levels.

Outcome measures included subjective reports of hunger before and after the intervention, as well as total energy intake immediately following an ad libitum breakfast. Additionally, changes in plasma glucose, insulin, 5‐HT, GLP‐1, DPP‐4, CCK, and ghrelin concentrations were tracked over 120 min post‐intervention. The study also incorporated the administration of ^13^C‐Na‐acetate, with breath sample analysis of the ^13^CO_2_/^12^CO_2_ ratio serving as an indicator of gastric emptying.

In the control group, a significant increase in subjective hunger was observed in the pre‐ and post‐intervention comparison (Figure SI‐2). In contrast, hunger levels remained stable following the pea protein interventions, regardless of which specific PPH was administered. Thus, both PPH1 and PPH2 interventions showed a reduced subjective feeling of hunger compared to the control intervention, suggesting a satiating effect with a similar magnitude for both hydrolysates.

However, the observed satiating effects of the PPH interventions could only be mirrored by a lower energy intake during the standardized ad libitum breakfast two hours post‐PPH2‐intervention compared to the control (Figure 2). For the PPH1 intervention, no significant difference in energy intake was observed compared to the control. To understand why only the more bitter‐tasting, less hydrolyzed PPH2 led to a reduced calorie intake, the two PPH interventions were normalized to the control and compared to each other in the following. This approach aims to identify differences between the two hydrolysates and clarify the underlying mechanisms.

The gastric emptying rate measures the speed of delivery of gastric contents into the duodenum [30]. Several studies have demonstrated that protein ingestion affects gastric emptying [18, 31, 32]. The study on gastric emptying revealed that gastric emptying was slower following the administration of PPH2 compared to the PPH1 intervention (Figure 3A,B). PPH2 exhibits greater bitterness than PPH1, which could be a factor in its slower gastric emptying. Wicks and colleagues [16] demonstrated that sham feeding of bitter substances reduces gastric emptying rate. In addition, the hydrolysates also vary in their degrees of hydrolysis. Staelens and colleagues [33] showed that the degree of hydrolysis also influences gastric emptying in healthy newborns, with a lower degree leading to delayed gastric emptying compared to protein formulas with a higher degree of hydrolysis. The results of both studies support these findings, as PPH2, which has a more intense bitter taste and a lower degree of hydrolysis (DH = 23%), delays gastric emptying compared to the less bitter, more hydrolyzed PPH1 (DH = 35%) and, therefore, induces a stronger effect on satiation.

Plasma levels of glucose and insulin (Figure SI‐3), as well as 5‐HT, CCK, and GLP‐1 (Figure SI‐4) remained unchanged across all interventions. Given that only 19 participants completed all four interventions, the small sample size may have limited the ability to detect significant effects on these plasma hormone levels.

On the other hand, the parameters ghrelin and DPP‐4 showed decreased plasma levels after the PPH1 intervention compared to the PPH2 intervention.

Ghrelin is a hormone that plays a crucial role in regulating hunger and food intake [7]. It is primarily produced in the stomach and, to a lesser extent, in the small intestine, pancreas, and brain. Its main functions include stimulating appetite, regulating meal timing, and influencing digestion and gastric motility [7]. Specifically, ghrelin is secreted when the stomach is empty, with levels rising before meals to signal the brain to initiate feelings of hunger. Ghrelin not only triggers hunger before meals but also helps regulate the timing of food intake. As mealtime approaches, ghrelin levels increase, prompting individuals to seek food. After eating, ghrelin levels decrease, curbing the sensation of hunger [34, 35]. Consuming protein typically leads to a greater and more sustained reduction in ghrelin levels than carbohydrates and fats. Protein‐rich meals promote satiety more effectively and suppress hunger for a longer period [36].

In our study, after evaluating the ghrelin plasma concentrations of the 19 participants, we observed that ghrelin levels decreased more after ingesting the less bitter, more highly hydrolyzed PPH1 compared to PPH2 (Figure 4A,B). Pupovac et al. [37] suggested that differences in peptide composition or the availability of active peptides in different hydrolysates may explain the varying feeding responses observed in mice. Häberer and colleagues [38] also reported that preloads of PPH reduced test‐meal size in rats, more so than isoenergetic amounts of intact pea protein. They indicated that certain proteins' short‐term satiating effects depend on their hydrolysis degree. This could also explain the lower ghrelin concentrations after the PPH1 intervention of our study, as the higher degree of hydrolysis in PPH1 may lead to faster plasma responses. Bioactive peptides are already present in the more highly hydrolyzed PPH1 and do not need to be released during gastric digestion, unlike PPH2, which has a lower degree of hydrolysis. To investigate the influence of bitter taste perception on blood markers related to satiety, the individual caffeine detection thresholds of each participant were determined. Ghrelin levels at individual time points, expressed as mean values, showed no significant differences between treatments in either the low or high caffeine detection threshold groups (Figure 4B,C). However, participants in the high caffeine detection threshold group exhibited higher AUC values for the more bitter PPH2 intervention compared to the less bitter PPH1 (*p* < 0.05, Figure 4F).

The second parameter found to be reduced in the blood plasma of the 19 male participants after ingestion of the less bitter, more hydrolyzed PPH1 was the enzyme DPP‐4. DPP‐4 was included in the human intervention study as a satiety marker due to its key regulatory role on the incretin hormone, GLP‐1 [39]. After food intake, GLP‐1 is released from intestinal L‐cells and exerts multiple satiety‐promoting effects, including delaying gastric emptying [40], stimulating insulin secretion [41] and signaling fullness to the brain [42]. However, GLP‐1 is rapidly degraded by the enzyme DPP‐4, thereby shortening its half‐life [43].

Recent research has identified food protein‐derived hydrolysates and peptides from various sources, such as egg, fish, milk, corn, and rice bran, that exhibit DPP‐4 inhibitory activity [44, 45]. In the presented study, plasma DPP‐4 levels were reduced after intervention with the less bitter‐tasting, more hydrolyzed PPH 1 solely (Figure 5A,B). This finding aligns with previous results, revealing an impact of the degree of hydrolysis of Atlantic salmon skin gelatin on DPP‐4 inhibition [46]. Thus, the higher degree of hydrolysis in PPH1, compared to PPH2, could be a key factor in the lower plasma DPP‐4 levels observed. Similar to ghrelin levels, DPP‐4 levels at individual time points, expressed as mean values, showed no significant differences between treatments in either the low or high caffeine detection threshold groups (Figure 5B,C). However, participants in the high caffeine detection threshold group again displayed higher AUC values for the PPH2 intervention compared to PPH1 (*p* < 0.05, Figure  5F), further supporting the notion that bitter taste is not the primary driver of satiety signals.

Although only the more bitter, less hydrolyzed PPH2 reduced energy intake from an ad libitum breakfast, we also observed blood markers in the less bitter, more hydrolyzed PPH1 group that indicate enhanced satiety. We hypothesized that the stronger bitterness combined with the lower degree of hydrolysis in PPH2 would both slow down gastric emptying and stimulate the release of satiety hormones into the blood. This hypothesis was confirmed for gastric emptying, as literature supports the notion that bitterness delays gastric emptying [16]. Additionally, the lower degree of hydrolysis influences gastric emptying [33] because the peptides in PPH2 require more extensive digestion [21], contributing to a prolonged retention time of the chyme in the stomach. However, for plasma hormone levels, the decreased DPP‐4 and ghrelin levels revealed after PPH1 intervention, compared to PPH2, suggest that hydrolysis degree may impact hormone release into the bloodstream. The higher degree of hydrolysis in PPH1 may yield bioactive peptides in the hydrolysate itself [20], potentially reducing hunger hormone and DPP‐4.

One explanation for the lack of a significant reduction in energy intake with PPH1 may be the delayed release of hormones into the bloodstream, as evidenced by the statistically significant reduction in DPP‐4 observed only after 120 min. In contrast, gastric emptying is slowed immediately upon ingestion of the PPH.

## 5 Conclusion

In summary, this human intervention study demonstrates that a bolus administration of 15 g of the more bitter, less hydrolyzed PPH2 during an oGTT reduced energy intake compared to the control group, which could not be demonstrated with a less bitter, higher hydrolyzed PPH1. When comparing the two PPHs, the more bitter, less hydrolyzed PPH2 slowed gastric emptying. Surprisingly, the less bitter and more extensively hydrolyzed PPH1 led to a greater reduction in ghrelin and DPP‐4 levels in blood plasma. This suggests that, in addition to bitterness, the degree of hydrolysis likely plays a more significant role in promoting satiety and satiation. Thus, both PPHs reduced hunger by targeting different mechanisms: PPH1 produced a faster response in satiety and hunger hormone levels in the blood, while PPH2 had a stronger impact on gastric emptying. These effects are likely due to the differing degrees of hydrolysis between the two PPHs.

## Ethics Statement

The human intervention study adhered to the principles of the Helsinki Declaration (1964) and its subsequent amendments. Ethical permission was approved by the ethics committee of the Technical University of Munich (reference number 2023‐610‐S).

## Consent

Informed consent was obtained from all participants included in the study. Participants were provided with an information sheet detailing the study procedure, potential risks, and participants' rights. Participants were explicitly informed of their right to decline participation in the study or to withdraw their consent at any stage without facing any negative consequences.

## Conflicts of Interest

The authors declare no conflicts of interest.

## Supporting information




**Supporting file 1**: mnfr70195‐sup‐0001‐SuppMat.doc

## Data Availability

Data are available on reasonable request from the authors
