# Supplementary material for: Randomized Controlled Trial: Effects of a Bitter‐Tasting Pea Protein Hydrolysate Intervention With Low Degree of Hydrolyzation on Energy Intake in Moderately Overweight Male Subjects
Source: Mol Nutr Food Res. 2025 Aug 8;69(21):e70195. doi: 10.1002/mnfr.70195 (PMC12581754; doi:10.1002/mnfr.70195)
Supplement: Supplementary file 1 — Supporting file 1: mnfr70195‐sup‐0001‐SuppMat.doc [file MNFR-69-e70195-s001.doc]

***Randomized controlled trial: Effects of a bitter-tasting pea protein hydrolysate intervention with low degree of hydrolyzation on energy intake in moderately overweight male subjects***

Katrin Gradl^1,2^, Sonja Sterneder^2^, Kristin Kahlenberg^2^, Beate Brandl^3^, Thomas Skurk^3^, Veronika Somoza^2,4,5*^

^1^TUM School of Life Sciences, Technical University of Munich, Alte Akademie 8, 85354 Freising, Germany

^2^*Leibniz Institute for Food Systems Biology at the Technical University of Munich, Lise-Meitner-Straße 34, 85354 Freising, Germany*

*^3^ZIEL - Institute for Food & Health, Weihenstephaner Berg 1, 85354 Freising, Germany*

^4^Chair of Nutritional Systems Biology, Technical University of Munich, Lise-Meitner-Straße 34, 85354 Freising, Germany

^5^*Department of Physiological Chemistry, Faculty of Chemistry, University of Vienna,* Josef-Holaubek-Platz 2*, 1090 Vienna, Austria*

**Correspondence to Veronika Somoza*

E-Mail:

Veronika Somoza: [v.somoza.leibniz-lsb@tum.de](mailto:v.somoza.leibniz-lsb@tum.de), telephone: +49 8161 71-2700

**KEYWORDS:** pea protein hydrolysate, human intervention study, satiety and satiation, gastric emptying


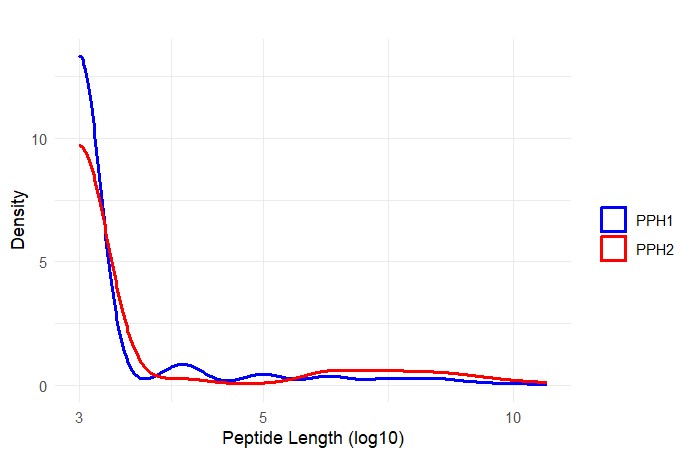


*Figure SI-1. Density plot of the distribution of the different peptide lengths in the PPH1 and PPH2. PPH1 has a higher degree of hydrolyzation with higher intensities of small tripeptides. PPH2 has a lower degree of hydrolyzation with higher density of peptides with 6 or more amino acids.*


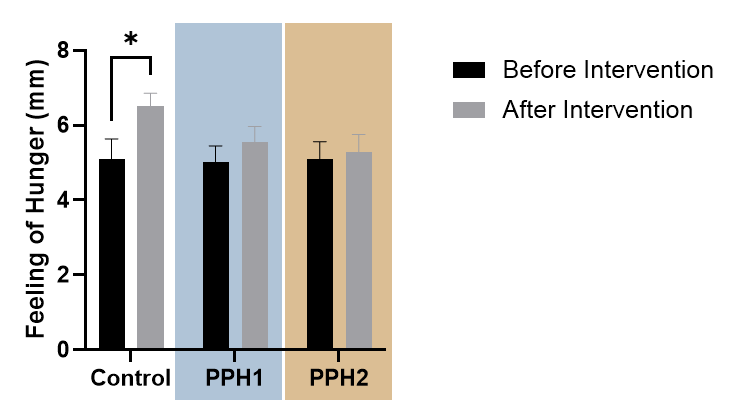


*Figure SI-2. Mean values of the feeling of hunger before and after 2h intervention determined by a 100 mm visual analogue scale (VAS). Statistics: 2way ANOVA with Tukey's multiple comparisons test: * = p ≤ 0.05.*

**
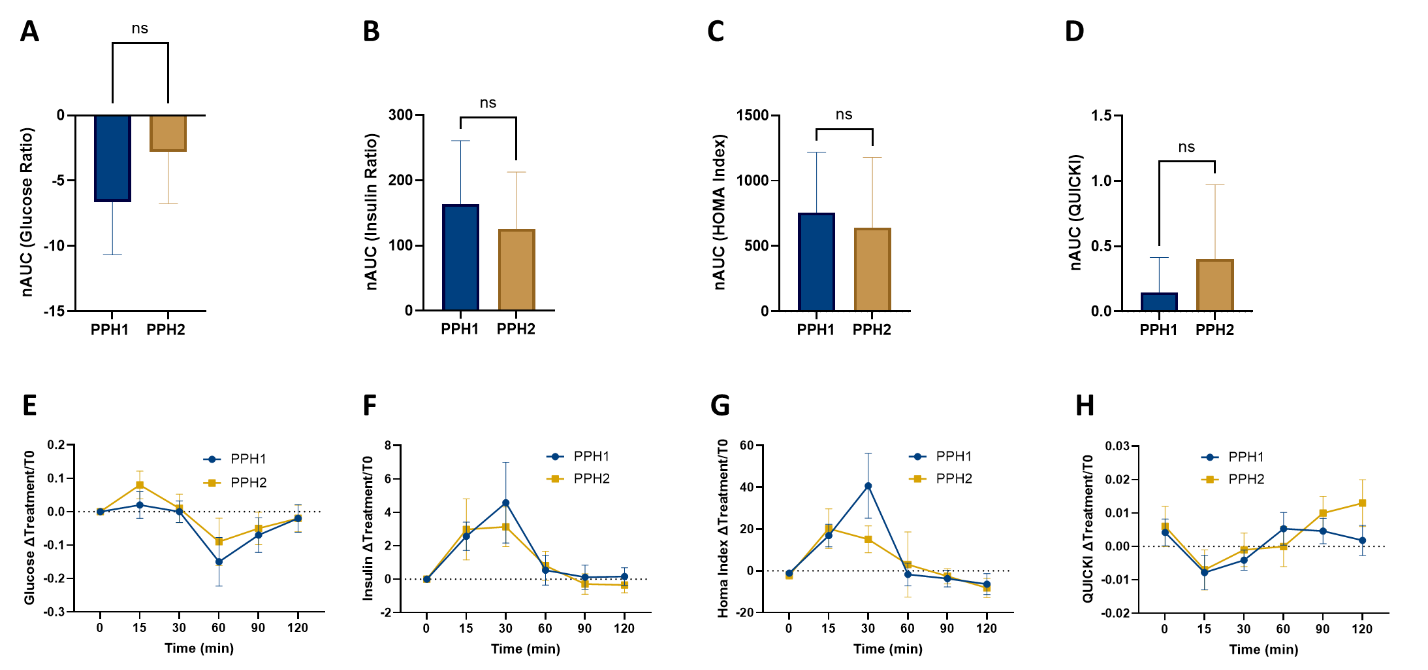
**

*Figure SI-3. Comparison of AUC of the time curves for (A) glucose, and (B) insulin blood plasma levels, (C) the HOMA index, and (D) the QUICKI after intervention with the hydrolysates PPH1 and PPH2 by calculating the ratio between the fasting concentrations and each time point. The mean values of 19 participants for each time point are shown for (E) glucose, (F) insulin, (G) the HOMA index, and (H) the QUICKI. Statistics: Wilcoxon test, significance values ​​are marked with ns = p > 0.05.*

**
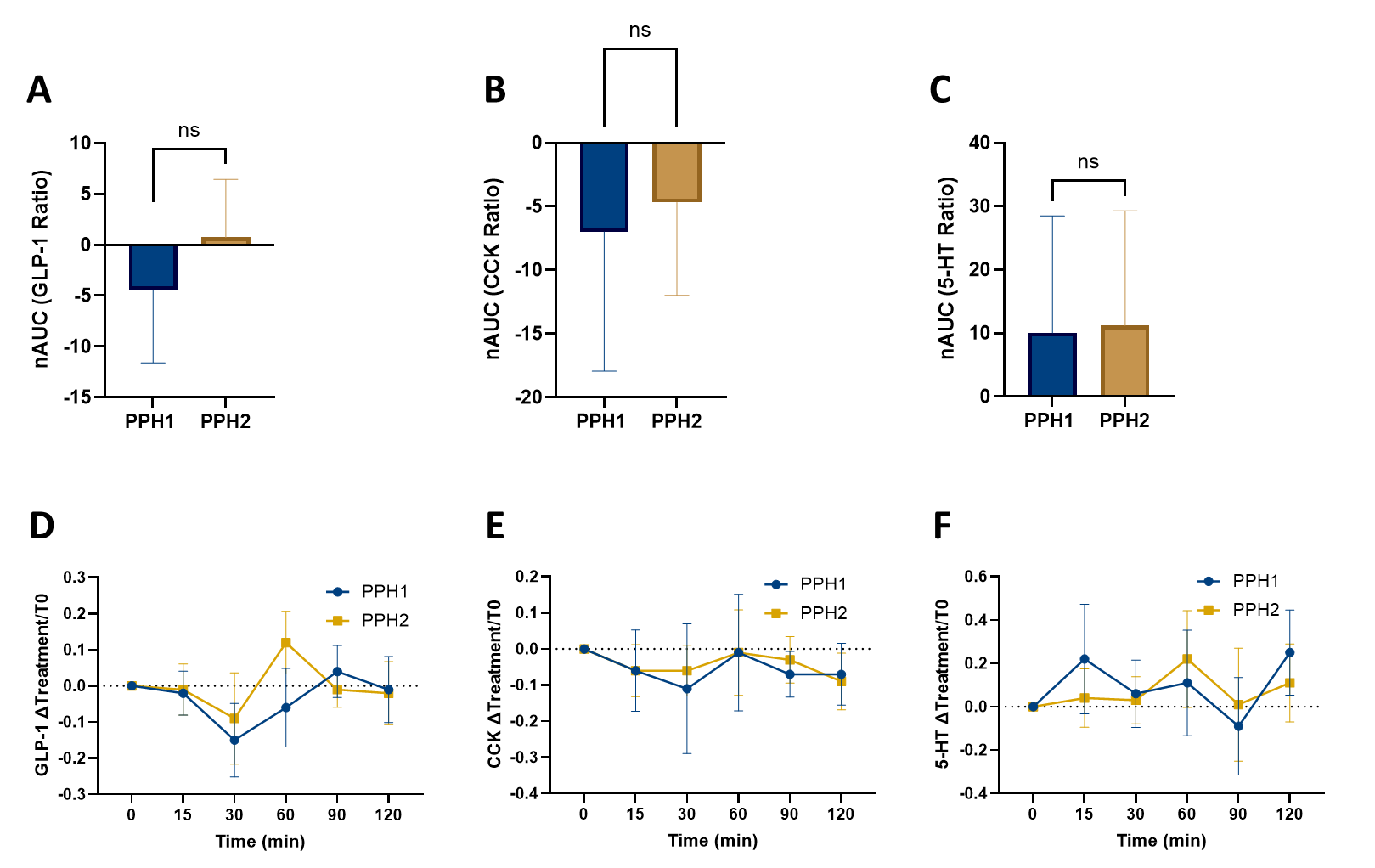
**

*Figure SI-4. Comparison of AUC of the time curves for (A) GLP-1, (B) CCK, and (C) 5-HT after intervention with the hydrolysates PPH1 and PPH2 by calculating the ratio between the fasting concentrations and each time point. The mean values of 19 participants for each time point are shown for (D) GLP-1, (E) CCK, and (F) 5-HT. Statistics: Wilcoxon test, significance values ​​are marked with ns = p > 0.05.*

*
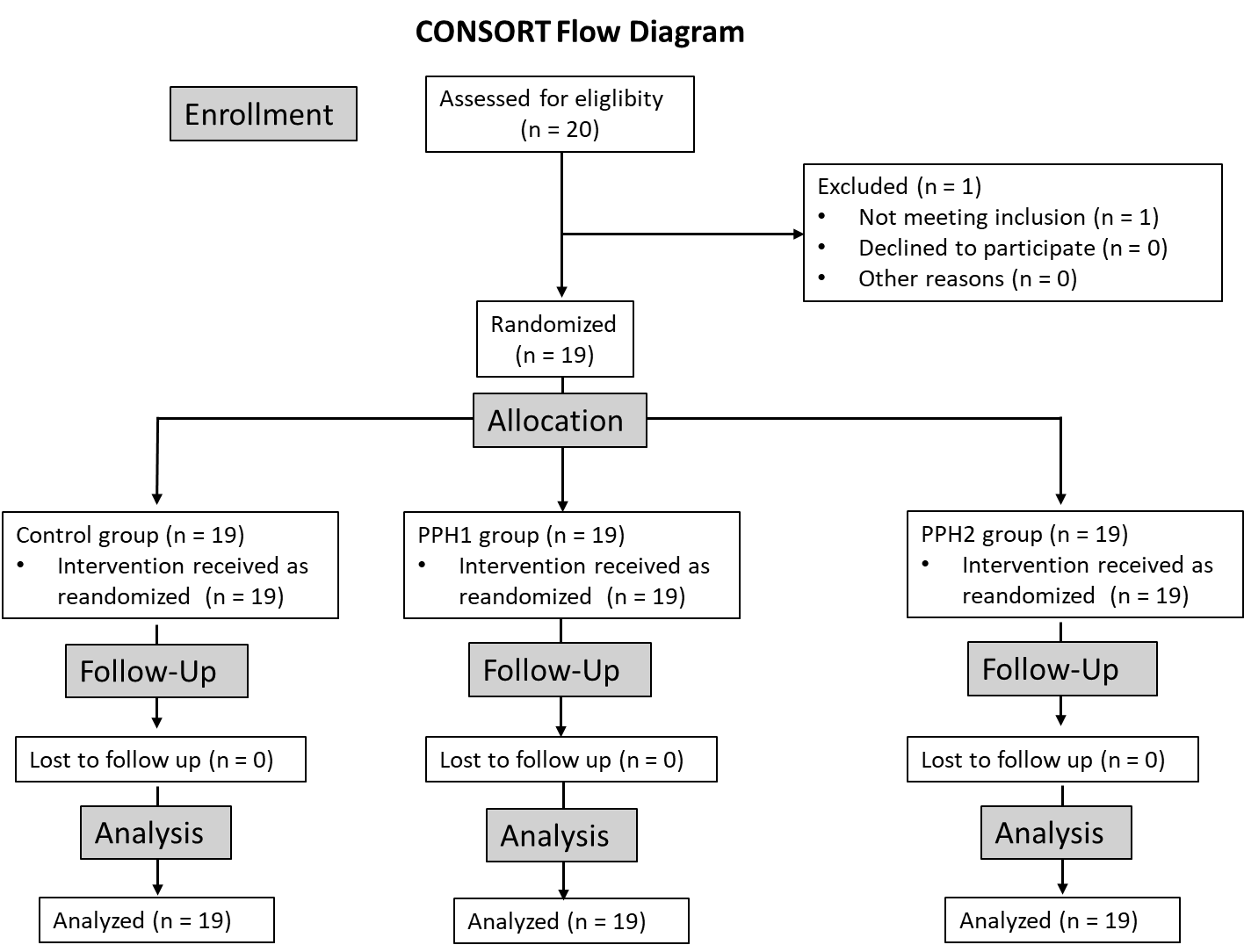
*

*Figure SI-5. CONSORT Flow-Chart.*
